# Wernicke’s Encephalopathy in a Child with Acute Lymphoblastic Leukemia

**DOI:** 10.4274/tjh.2016.0044

**Published:** 2017-03-01

**Authors:** Hande Kızılocak, Gül Nihal Özdemir, Gürcan Dikme, Zehra Işık Haşıloğlu, Tiraje Celkan

**Affiliations:** 1 İstanbul University Cerrahpaşa Faculty of Medicine, Department of Pediatric Hematology-Oncology, İstanbul, Turkey; 2 İstanbul University Cerrahpaşa Faculty of Medicine, Department of Radiology, İstanbul, Turkey

**Keywords:** Wernicke’s encephalopathy, Thiamine deficiency, Pediatric leukemia

## TO THE EDITOR,

We read with great interest the article “A rare complication developing after hematopoietic stem cell transplantation: Wernicke’s encephalopathy” by Solmaz et al. [1]. Wernicke’s encephalopathy (WE) is an acute syndrome requiring emergent treatment to prevent death and neurologic morbidity [2]. While most often associated with alcoholism, WE also occurs in the setting of prolonged intravenous feeding without adequate thiamine supplementation, prolonged starvation or unbalanced nutrition, gastrointestinal surgery, systemic malignancy, and transplantation [3]. The classic triad of WE includes encephalopathy, oculomotor dysfunction, and gait ataxia. In their article, Solmaz et al. reported a patient who developed WE following hematopoietic stem cell transplantation (HSCT) and they concluded that this was due to prolonged total parental supplementation and lack of thiamine supplementation. The only other suggested cause was the use of busulfan in the conditioning regimen. In the literature there is a link of WE to HSCT, malignancies, or chemotherapies. Here we report a new patient who developed WE during acute lymphoblastic leukemia (ALL) treatment.

**Figure 1:**
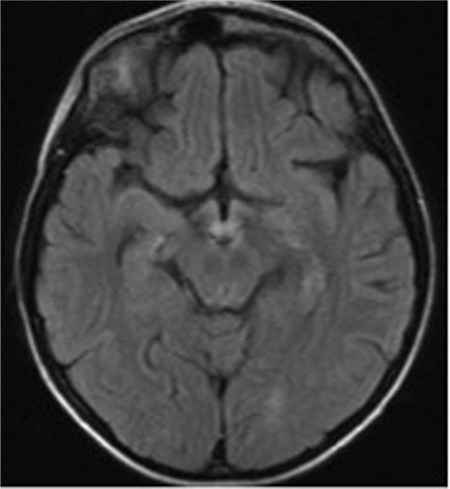
Increased signal in the bilateral thalamic pulvinar and mammillary bodies in the axial fluid-attenuated inversion recovery sequence.

A 13-year-old female patient diagnosed with intermediate risk group ALL developed severe neutropenia after a high-dose methotrexate block and oral Purinethol (BFM protocol M). Ceftazidime and fluconazole treatment was started due to fever. After 3 days the patient had poor oral intake and received total parenteral nutrition (TPN) containing protein and dextrose. On the 6^th^ day of TPN she had fever, abdominal pain, nausea, and bilious vomiting. Her abdominal ultrasound revealed typhlitis. Ceftazidime-fluconazole treatment was switched to meropenem and L-amphotericin and oral intake was stopped. On the 8^th^ day of TPN, the patient developed confusion, altered mental status, horizontal nystagmus, and lateral gaze paralysis in the right eye. Her brain computed tomography (CT) was normal. However, brain magnetic resonance imaging (MRI) showed increased signal in the bilateral thalamic pulvinar and mammillary bodies in the axial fluid-attenuated inversion recovery (FLAIR) sequence (Figure 1). These were concluded to be classic findings of WE [4]. Intramuscular thiamine at 200 mg three times a day for the first 3 days (600 mg/day total), 100 mg two times a day for the next 3 days (200 mg/day total), and 100 mg thiamine daily for the last 3 days was given. A rapid improvement of neurologic symptoms was observed on the third day of thiamine treatment. The patient’s thiamine level was 55 mg/L and 125 mg/L before and after the treatment, respectively (normal range: 25-75 mg/L). She was discharged from the hospital with good oral intake and normal neurological examination.

WE is primarily a clinical diagnosis. Response to treatment may be diagnostic. The sensitivity and specificity of serum thiamine level in symptomatic patients is unclear, as the blood level may not reflect the brain thiamine level. A normal blood thiamine level, as in our patient, does not exclude the possibility of WE with MRI findings [5]. MRI is more sensitive than CT in WE [6]. In conclusion, all at-risk patients with undiagnosed altered mental status, oculomotor disorders, or ataxia must be evaluated for WE. Further studies are needed for examining the possible role of chemotherapeutics in the development of WE.

## References

[1] Solmaz S, Gereklioğlu Ç, Tan M, Demir Ş, Yeral M, Korur A, Boğa C, Özdoğu H (2015). A rare complication developing after hematopoietic stem cell transplantation: Wernicke’s encephalopathy. Turk J Hematol.

[2] Park SW, Yi YY, Han JW, Kim HD, Lee JS, Kang HC (2014). Wernicke’s encephalopathy in a child with high dose thiamine therapy. Korean J Pediatr.

[3] Parkin AJ, Blunden J, Rees JE, Hunkin NM (1991). Wernicke-Korsakoff syndrome of nonalcoholic origin. Brain Cogn.

[4] Beh SC, Frohman TC, Frohman EM (2013). Isolated mammillary body involvement on MRI in Wernicke’s encephalopathy. J Neurol Sci.

[5] Davies SB, Joshua FF, Zagami AS (2011). Wernicke’s encephalopathy in a non-alcoholic patient with a normal blood thiamine level. Med J Aust.

[6] Elefante A, Puoti G, Senese R, Coppolo C, Russo C, Tortoro F, Divitiis O, Brunetti A (2012). Non-alcoholic acute Wernicke’s encephalopathy: role of MRI in non typical cases. Eur J Radiol.

